# Prevalence of heartworm in dogs and cats of Madrid, Spain

**DOI:** 10.1186/s13071-017-2299-x

**Published:** 2017-07-26

**Authors:** José Alberto Montoya-Alonso, Rodrigo Morchón, Yaiza Falcón-Cordón, Soraya Falcón-Cordón, Fernando Simón, Elena Carretón

**Affiliations:** 1 Internal Medicine, Faculty of Veterinary Medicine, Research Institute of Biomedical and Health Sciences (IUIBS), University of Las Palmas de Gran Canaria, Las Palmas de Gran Canaria, Spain; 2 Laboratory of Parasitology, Faculty of Pharmacy, Institute of Biomedical Research of Salamanca (IBSAL), University of Salamanca, Salamanca, Spain

**Keywords:** Feline, Canine heartworm, *Dirofilaria immitis*, Vector-borne diseases, Prevalence, Epidemiology, Spain

## Abstract

**Background:**

*Dirofilaria immitis* causes heartworm disease, a chronic and potentially fatal cardiopulmonary disease which mainly affects dogs and cats. It is present in most of Spain, due to favourable climatic factors. Madrid, located in the centre of the Iberian Peninsula, is the most highly populated city in the country. There is a lack of current data on canine heartworm and there are no published epidemiological data regarding feline heartworm in this region, therefore the aim of this study was to assess the prevalence and current distribution of canine and feline dirofilariosis in the province of Madrid.

**Methods:**

Serum samples from 1716 dogs and 531 cats, from animals living in the metropolitan area of Madrid and adjacent areas, were studied. All the samples, either from cats and dogs, were tested for circulating *D. immitis* antigens using a commercial immunochromatographic test kit. Furthermore, to establish the seroprevalence of heartworm infection in cats, serological techniques for anti-*D. immitis* and anti-*Wolbachia* antibody detection were used.

**Results:**

Prevalence of *D. immitis* in the canine population of Madrid was 3%, showing an increase in comparison to previous data. The presence of heartworm in the city centre could be influenced by the presence of Urban Heat Islands, while the positive dogs from metropolitan and adjacent areas were mainly located under the influence of rivers. Regarding cats, 0.2% were positive to the antigens test and 7.3% were seropositive to both anti-*D. immitis* and *Wolbachia* surface protein antibodies, which demonstrate the presence of feline heartworm in Madrid. Seropositive cats were present in the same areas where positive dogs were found. Indoor/outdoor cats showed the highest seroprevalence whereas the lowest corresponded to indoor cats, demonstrating that prophylactic treatments should be carried out regardless of lifestyle. Infection was found in 2.2% of dogs and 6.7% of the cats < 1 year-old, which indicates that early preventive campaigns in puppies and kittens should be implemented.

**Conclusions:**

The results point to the need for adequate prophylactic measures through the administration of macrocyclic lactones in animals living in Madrid. Veterinarians should be aware of the presence of this disease and include heartworm in the differential diagnosis when a pet presents with symptoms compatible with *D. immitis*.

## Background

Heartworm disease is caused by *Dirofilaria immitis* and is a chronic, progressive and potentially fatal disease for the infected animals. The main hosts are dogs and cats, although infection has been reported in other carnivores, including wild canines as well as domestic and wild felids. Furthermore, humans can be infected since it is a zoonotic disease [1]. The transmission occurs through the bite of the species of culicid mosquitoes belonging to the genera *Culex*, *Aedes* and *Anopheles*, among others. Thus, prevalences are influenced by the presence and abundance of vectors, depending on climatic factors such as temperature and humidity [1, 2].

Animal dirofilariosis has been extensively studied in Europe. The geographical location of Spain, in southern Europe, situates the Iberian Peninsula in the endemic area of dirofilariosis on the continent [3, 4]. In Spain, the infection is present in most of the territory, the highest prevalences being found in the southwest, Mediterranean coast and irrigated areas of inland Spain and in the Canary Islands [5–7].

The province of Madrid is in the centre of the Iberian Peninsula. The altitude varies from 550 to 700 m in most of the territory, except in the northernmost part of the province, where the mean height mainly varies from 1000 to 1700 m, reaching above 2000 m at some points. Many river basins cross the territory from north to south, carrying the water flow to the River Tajo, which crosses the province in the southern part of the region. The province presents cold winters and mild summers in the northern part of the territory, while the rest of the territory is characterized by cold winters and hot summers; these are the driest seasons, while rains are more frequent in autumn and spring.

The city of Madrid is the capital of the province, and is also the capital of Spain. It is the most highly populated city of the country, as only the centre of Madrid has a census of over 3 million people, 275,000 dogs and 65,000 cats [8, 9]. The city is included within the metropolitan area, which surrounds the city representing the most highly populated metropolitan area of Spain, with an estimated population above 7 million and the fifth most populated metropolitan area of Europe.

Published studies reported canine dirofilariosis of 1.1 and 1.9%, in the province of Madrid, between 1987 and 1990 [10, 11]. No studies followed these data until 2013, when a study reported prevalences of 2.3% in a wide area of central Spain which enclosed Madrid and Toledo [12]. There are no epidemiological data published regarding cats.

There is a lack of current epidemiological data of animal heartworm focusing on the province of Madrid. Therefore, the aim of this study was to assess the prevalence and current distribution of canine and feline dirofilariosis in the metropolitan area of Madrid and adjacent areas.

## Methods

**Fig. 1:**
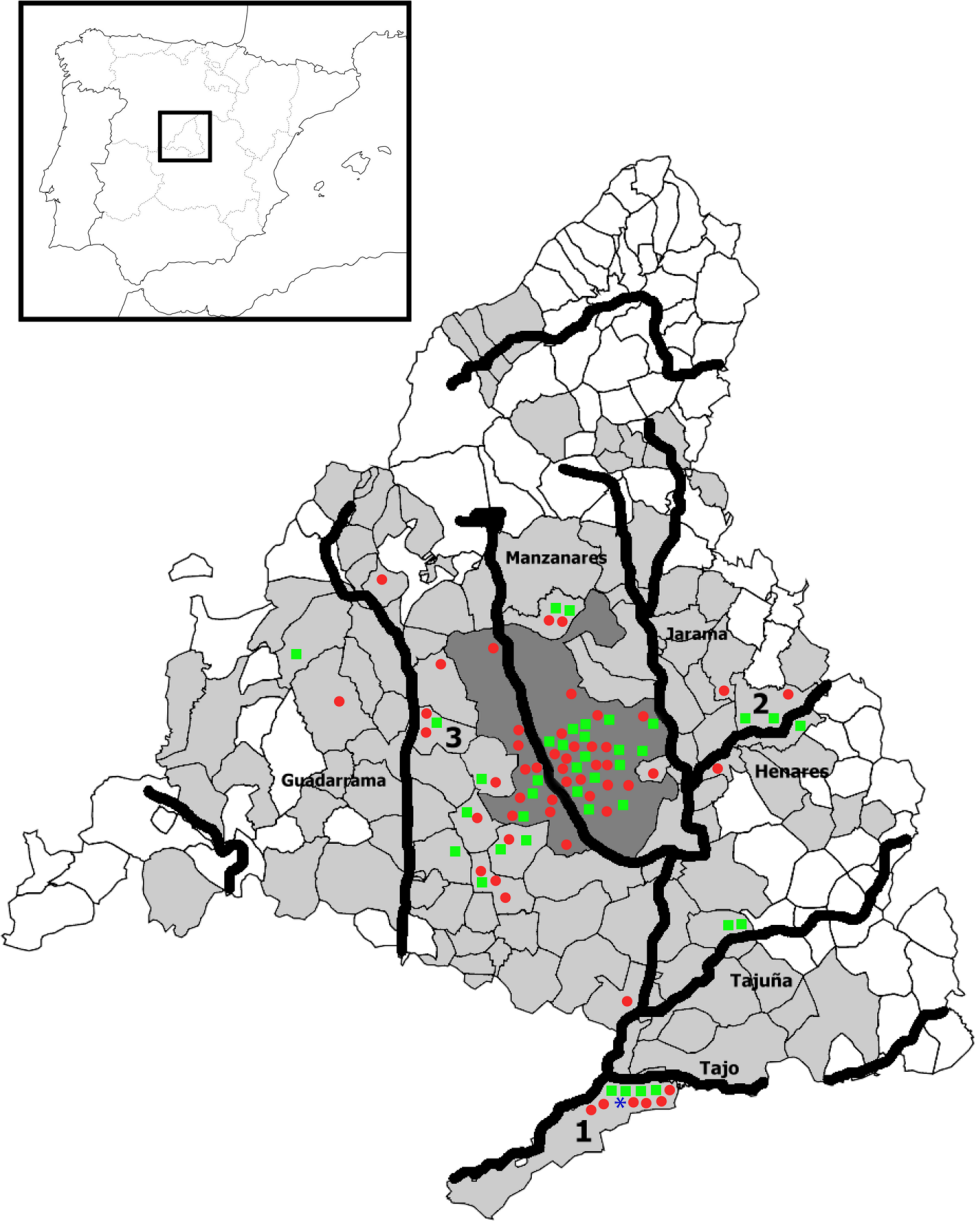
Distribution of canine and feline heartworm in the province of Madrid (Spain). The dark grey area corresponds to the city of Madrid, while the pale grey area corresponds to the metropolitan and adjacent area included in the study. The main rivers are illustrated as thick black lines. The positive animals are marked as follows: *red circles* (heartworm positive dogs); *green squares* (antibodies-seropositive cats); *blue asterisk* (antigens-positive cat). The municipalities with higher prevalences and seroprevalences are indicated as: 1, Aranjuez; 2, Alcalá de Henares; and 3, Majadahonda

The present study included 1716 dogs and 531 cats presented to veterinary clinics between March 2015 and September 2016. The samples were collected at 20 veterinary centres located in the studied area from animals living in the metropolitan area of Madrid and adjacent areas, including 98 municipalities in 218 different postal codes (Fig. 1).

The inclusion criteria of animals were: being over 6 months of age; not having travelled outside the area of interest of the study; never having received treatment for heartworm disease; and no previous history of heartworm infection. A complete record was kept for each animal, including identification (age, sex and breed), clinical history, and demographic data.

Of the dogs included, 46.7% were females and 53.3% were males. By breed, the most highly represented were mongrel dogs (26.3%), followed by Yorkshire Terriers (8.6%), Golden Retrievers (6.9%), Cocker Spaniels (5.7%), Boxer (5.3%), French Bulldog (4.8%), German Shepherd (4.1%) and 63 other breeds (38.3%). Regarding the cats studied, 51.4% were females and 48.4% were males; by breed, the European shorthair was the most highly represented (65.3%), followed by Persian cats (14.7%), Siamese cats (12.2%) and other 15 breeds (7.8%). The cats were further classified as indoor (57.6%), outdoor (12.6%) or indoor/outdoor (at least 1–50% of the time spent outdoors) (29.8%). The age ranges for dogs and cats are described in Table 1. The study included 845 dogs and 312 cats living in the city of Madrid while 871 dogs and 219 cats were living in the rest of the studied area (metropolitan and adjacent area).

**Table 1:** Dogs (*n*) and cats (*n*) studied by age ranges, as well as prevalence of heartworm (%) by each group of age

Age range	Dogs (*n*)	Canine prevalence (%)^a^	Cats (*n*)	Feline seroprevalence (%)^b^
< 1 year	45	2.2	15	6.7
1–3 years	320	3.7	78	1.3
3–6 years	393	3.6	213	8.0
6–9 years	429	3.3	88	11.4
9–12 years	396	2.0	74	5.4
> 12 years	133	2.3	63	9.5

^a^Based on detection of circulating *D. immitis* antigens

^b^Based on serological techniques for anti-*D. immitis* and anti-*Wolbachia* antibody detection (considered seropositive when anti-*D. immitis* and anti-*Wolbachia* surface proteins antibodies presented jointly)

Blood samples were collected from the cephalic or jugular vein, placed in 3 ml serum tubes and centrifuged. Serum was kept at -20 °C until tests were performed. All the samples, either from cats and dogs, were tested for circulating *D. immitis* antigens using a commercial immunochromatographic test kit (Uranotest Dirofilaria®, Urano Vet SL, Barcelona, Spain) according to the manufacturer’s instructions.

Furthermore, to establish the seroprevalence of heartworm infection in cats, serological techniques for anti-*D. immitis* and anti-*Wolbachia* antibody detection were also used, as described by Morchón et al. [13] with some modifications. In brief, the plates were coated with 0.8 μg of *D. immitis* somatic antigen and *Wolbachia* surface protein (WSP). Serum samples were prepared at 1/100 for anti-*D. immitis* serum antibodies and 1/40 for anti-WSP antibody detection. Anti-feline IgG antibody, horseradish peroxidase-labelled (Kirkegaard and Perry Laboratories, Gaithersburg, MD, USA), was applied at 1/4000 dilution. The optical densities were measured in an Easy-Reader (Bio-Rad Laboratories, Hercules, CA, USA) at 492 nm. Cut-off points of ELISA *D. immitis* 0.8 and ELISA WSP 0.6 were obtained as arithmetic mean optical density ± 3 standard deviations of serum of clinically healthy cats. Cats were considered seropositive when anti-*D. immitis* and anti-WSP antibodies presented jointly [7, 14–16].

Data were analysed using SPSS Base 20.0 software for Windows (SPSS Inc./IBM, Chicago, IL, USA). Descriptive analysis of the considered variables was carried out considering the proportions of the qualitative variables. *χ* ^2^ to compare proportions was performed. In all cases, the significance level was established at *P* < 0.05.

## Results

The prevalence of *D. immitis* in the canine population of the studied area of Madrid was 3% (52/1716). There were not significant differences in the prevalences between males (3.2%) and females (2.9%) (*χ* ^2^ = 0.006, *df* = 1, *P* = 0.939). By breed, mongrel dogs showed higher but not significantly different prevalences (4.2%) when compared with pure-breed dogs (2.6%) (*χ* ^2^ = 2.902, *df* = 1, *P* = 0.088). By age ranges, the highest prevalences were found in dogs from 1 to 6 years, and significant differences were only observed between dogs betwee 9–12 year-old compared with dogs between 1 and 6 year-old (*χ* ^2^ = 2.753, *df* = 5, *P* = 0.738) (Table 1).

Antigens test were positive in 0.2% (1/531) of the cats, while 7.3% (39/531) of cats were seropositive to both anti-*D. immitis* and WSP antibodies. There were no statistically significant differences between females (7.7%) and males (7.0%) (*χ* ^2^ = 0.172, *df* = 2, *P* = 0.918). By breed, the European shorthair presented a seroprevalence of 7.5% while the mean seroprevalence in the other breeds was 7.1% (*χ* ^2^ = 0.036, *df* = 1, *P* = 0.850). Indoor/outdoor cats showed the highest seroprevalence (11.4%), followed by outdoor cats (10.4%) while the lowest corresponded to indoor cats (4.6%) (*χ* ^2^ = 8.201, *df* = 2, *P* = 0.017). By age ranges, the highest seroprevalence was found in cats between 6 and 9 year-old; no significant differences were found between all groups of age (*χ* ^2^ = 7.265, *df* = 5, *P* = 0.202) (Table 1).

The prevalence of heartworm infection in the city of Madrid in the canine population was 3.3%. By sex, both males and females showed the same prevalence (3.3%). There were positive dogs in 15 of the 21 districts of the city. The prevalence in the metropolitan and adjacent areas was 2.7% (2.4% in females and 3% in males); the distribution was not uniform, the positive dogs being mainly located in the area of influence of the major rivers. By municipalities, the highest prevalences were observed in Aranjuez (10.0%), Majadahonda (6.4%) and Alcalá de Henares (5.9%), all of them influenced by rivers (Fig. 1).

When the cats were analysed, the seropositivity in the city of Madrid was 6.1%. By sex, the seroprevalence was 7.6% in females and 4.3% in males. There were seropositive cats present in 11 of the 21 districts of the city.

The seroprevalence was higher in the metropolitan and adjacent areas, this being 9.1% (7.7% in females and 10.3% in males). Positive animals were mainly found in areas close to rivers. By municipalities, the highest seroprevelances were observed in Alcalá de Henares (27.3%), Aranjuez (20.8%) and Majadahonda (11.1%), all of them under the influence of river basins. The only cat positive to the antigens test corresponded to a female European shorthair, living outdoors in Aranjuez (Fig. 1).

## Discussion

The present study reports the presence of heartworm infection in the pet population of Madrid and establishes the current canine prevalence at 3%. Previous reported data on heartworm infection in dogs from Madrid between 1987 and 1990 varied from 1.1 to 1.9% [10, 11]. More recent data reported a prevalence of 2.3% in 2013, although the prevalence was defined to a wide area of central Spain which enclosed Madrid and Toledo with a relatively low sample size [12]. Considering previous data, it can be considered that the prevalence of canine heartworm in Madrid has increased.

There are several climatic conditions that could favour the transmission of dirofilariosis in the studied area. The province of Madrid is under the influence of several river basins, which provides a humid environment appropriate for the development of the mosquitoes. The results from the study show how currently positive dogs outside the city centre are mainly concentrated in areas under the influence of the rivers Tajo, Jarama, Henares and Guadarrama. The prevalence detected in Aranjuez is especially striking; the area is a small urbanized municipality under the influence of the River Tajo, where dogs live outdoors more frequently than in urban areas. An increase in the prevalence from that 6.8% reported in 1990 [10] to the current 10% was also observed. Furthermore, the city of Madrid is also under the influence of the River Manzanares, which crosses the city centre from west to east.

The high levels of urbanization of the city of Madrid and the closest metropolitan area are under the influence of the phenomenon called Urban Heat Island (UHI). As buildings and asphalt retain heat during the day which is re-emitted towards the atmosphere, the temperature increases several degrees inside the city creating microclimates [17, 18]. In the case of Madrid, multiple studies on UHI have been carried out during the last two decades [19, 20]. Furthermore, human activity increases the density of mosquitoes and develops a suitable environment for its proliferation due to an increase in the provision of water sources and vegetation which can be found in the presence of parks and green urban areas. Since the mosquitoes can reproduce in small containers with water (flowerpots, ponds, fountains), all these factors favour the climatic conditions for the development of heartworm larvae in mosquito vectors during the cold months of the year. This phenomenon was also observed in Barcelona, another large city in Spain [16, 21]. In the present study, the prevalence observed in the city centre was 3.3%. A previous study reported a prevalence of 0.6% in the urban area of Madrid in 1990 [10], therefore it can be concluded that the prevalence in the city centre has also increased. This could be due to the exacerbation of the phenomenon of the UHI, a greater presence of green urban areas, a higher density of dogs in the city and a low incidence chemoprophylaxis. Dogs living in urban areas are not safe from heartworm infection and should receive adequate prophylaxis with macrocyclic lactones, as concluded in a similar study in Barcelona [21].

Geographical information systems (GIS) can predict the distribution and epidemiological behaviour of dirofilariosis in different territories [3, 21–23] based on climatic, ecological and many other data related to the developing requirements of parasites and vectors. A GIS research on the general prediction for the distribution of dirofilariosis in Spain showed that the area included in this study is at high risk of *D. immitis* infection [23]; among other risk factors, due to the existence of irrigated crops which increases the transmission risk, providing excellent habitats for mosquito breeding.

Regarding cats, the present results report the first epidemiological data on feline *D. immitis* and demonstrate the presence of feline heartworm in Madrid. To date, in Spain epidemiological studies in heartworm infection in cats have been previously reported only in the Canary Islands and in Barcelona [7, 14, 16]. The results showed that 0.2% of cats were positive to *D. immitis* antigens in comparison with 7.3% seropositivity to antibodies. This may be due to the fact that the sensitivity of antigen testing is relatively low in cats, and because these only detect adult and female worms. Therefore, a negative result does not rule out an infection from male worms, pre-adult worms or a single female adult worm, most of which are common in cats [24, 25]. On the other hand, antibody tests detect antibodies produced by the host in response to infection, which therefore can remain positive for a long time after the death of the parasite and do not differentiate a current or a past infection [24, 26]. Given the natural resistance of the feline host to heartworm infection, it is estimated that the feline dirofilariosis is 5–20% of that of the canine population in the same area [27, 28], and feline heartworm reported in the present study is within these ranges.

The results showed that cats living indoor/outdoor and indoor presented higher seroprevalences, presumably because they are more exposed to mosquito bites. However, 4.6% of indoor cats showed *D. immitis* antibodies demonstrating that an indoor lifestyle does not protect cats from infection [16, 29]. Cats do not usually receive chemoprophylaxis and therefore the risk of infection is higher in this species than in dogs. Seropositive cats were present in the same areas where positive dogs were found, influenced by the same climatic conditions. The seroprevalence is higher in cats from metropolitan and adjacent areas when compared with the city centre; probably one of the reasons may be because cats living in those municipalities spend time outdoors more frequently. Therefore, adequate prophylactic treatments with macrocyclic lactones should be carried out in this species, regardless of lifestyle.

Although not statistically significant, mongrel dogs showed higher prevalences when compared with pure-breed dogs, as reported in other studies [21]. Regarding cats, no significant differences were observed between European shorthair and all the other breeds. By age, the highest prevalence was found in dogs from 1 to 3 years followed by dogs from 3 to 6 years; other studies showed the highest prevalences in older dogs [14, 23]. In cats, a great variability in the prevalences by age ranges can be observed when the results were compared with other studies [7, 14–16, 29]. It is noteworthy to mention that 2.2% of dogs < 1 year-old were infected and 6.7% of the cats were seropositive, which indicates that early preventive campaigns in puppies and kittens with macrocyclic lactones should be carried out.

## Conclusions

The results show the presence of canine and feline heartworm in the province of Madrid, in the centre, metropolitan and adjacent areas and an increase in the prevalence compared to previous studies. These results point to the need for prophylactic measures with the administration of macrocyclic lactones to control the disease and to avoid zoonotic infections. Veterinary clinicians should be aware of the presence of this disease and include heartworm in the differential diagnosis when a pet presents with respiratory signs or symptoms compatible with *D. immitis*.

## Abbreviations

GIS: Geographical information systems
UHI: Urban Heat Island
WSP: *Wolbachia* surface protein

## References

[1] Simón F, Siles-Lucas M, Morchón R, González-Miguel J, Mellado I, Carretón E (2012). Human and animal dirofilariasis: the emergence of a zoonotic mosaic. Clin Microbiol Rev.

[2] Cancrini G, Magi M, Gabrielli S, Arispici M, Tolari F, Dell’Omodarme M, et al. Natural vectors of dirofilariasis in rural and urban areas of the Tuscan region, central Italy. J Med Entomol. 2006;43(3):574–79. 10.1603/0022-2585(2006)43[574:nvodir]2.0.co;2 16739418

[3] Genchi C, Rinaldi L, Mortarino M, Genchi M, Cringoli G (2009). Climate and *Dirofilaria* infection in Europe. Vet Parasitol.

[4] Genchi C, Kramer LH, Rivasi F (2011). Dirofilarial infections in Europe. Vector Borne Zoonotic Dis.

[5] Montoya JA, Morales M, Juste M C, Corbera JA. Heartworm (*Dirofilaria immitis*) infection in dogs: current update in Spain. In: Genchi C, Rinaldi L, Cringoli G, editors. *Dirofilaria immitis* and *D. repens* in dog and cat and human infections. Naples: Rolando Editore; 2007. pp. 175–80.

[6] Morchón R, Carretón E, González-Miguel J, Mellado-Hernández I (2012). Heartworm disease (*Dirofilaria immitis*) and their vectors in Europe - new distribution trends. Front Physiol.

[7] Montoya-Alonso JA, Carretón E, Morchón R, Silveira-Viera L, Falcón Y, Simón F (2016). The impact of the climate on the epidemiology of *Dirofilaria immitis* in the pet population of the Canary Islands. Vet Parasitol.

[8] Census of domestic animals of the city of Madrid, by district. Ilustre Colegio Oficial de Veterinarios de Madrid and Ayuntamiento de Madrid. 2016. http://datos.madrid.es/egob/catalogo/207118-0-censo%20animales.xlsx Accessed 17 Mar 2017.

[9] [Municipal Register of Inhabitants City of Madrid]. Área de Gobierno de Economía y Hacienda Subdirección General de Estadística. Ayuntamiento de Madrid. 2016. http://www.madrid.es/UnidadesDescentralizadas/UDCEstadistica/Nuevaweb/Demograf%C3%ADa%20y%20poblaci%C3%B3n/Cifras%20de%20poblaci%C3%B3n/PMH/Informe/Informe_PMH%202016.pdf Accessed 17 Mar 2017.

[10] Rojo-Vázquez FA, Valcárcel F, Guerrero J, Gómez M (1990). Prevalencia de la dirofilariosis canina en cuatro áreas geográficas de España. Med Vet.

[11] Ortega-Mora LM, Gomez-Bautista M, Rojo-Vazquez F, Rodenas A, Guerrero J (1991). A survey of the prevalence of canine filariasis in Spain. Preventive Vet Med.

[12] Miró G, Montoya A, Roura X, Gálvez R, Sainz A (2013). Seropositivity rates for agents of canine vector-borne diseases in Spain: a multicentre study. Parasit Vectors.

[13] Morchón R, Ferreira AC, Martín-Pacho JR, Montoya-Alonso JA, Mortarino M, Genchi C (2004). Specific IgG antibody response against antigens of *Dirofilaria immitis* and its *Wolbachia* endosymbiont bacterium in cats with natural and experimental infections. Vet Parasitol.

[14] Montoya-Alonso JA, Carretón E, Corbera JA, Juste MC, Mellado I, Morchón R, et al. Current prevalence of *Dirofilaria immitis* in dogs, cats and humans from the island of Gran Canaria. Spain: Vet Parasitol; 2011;176(4):291–4. 10.1016/j.vetpar.2011.01.011 21310532

[15] Vieira L, Silvestre-Ferreira AC, Fontes-Sousa AP, Balreira AC, Morchón R, Carretón E (2015). Seroprevalence of heartworm (*Dirofilaria immitis*) in feline and canine hosts from central and northern Portugal. J Helminthol.

[16] Montoya-Alonso JA, Carretón E, García-Guasch L, Expósito J, Armario B, Morchón R (2014). First epidemiological report of feline heartworm infection in the Barcelona metropolitan area (Spain). Parasit Vectors.

[17] Oke TR (1982). The energetic basis of the urban heat island. Q J Roy Meteor Soc.

[18] Gago EJ, Roldan J, Pacheco-Torres R, Ordóñez J (2013). The city and urban heat islands: a review of strategies to mitigate adverse effects. Renew Sust Energ Rev.

[19] Yagüe C, Zurita E, Martínez A (1991). Statistical analysis of the Madrid urban Heat Island. Atmos Environ.

[20] Sobrino JA, Sòria G, Oltra-carrió R, Jiménez-Muñoz JC, Romaguera M, Cuenca J (2009). DESIREX 2008: urban Heat Island analysis in the City of Madrid. Revista de Teledetección.

[21] Montoya-Alonso JA, Carretón E, Simón L, González-Miguel J, García-Guasch L, Morchón R, Simón F (2015). Prevalence of *Dirofilaria immitis* in dogs from Barcelona: validation of a geospatial prediction model. Vet Parasitol.

[22] Mortarino M, Musella V, Costa V, Genchi C, Cringoli G, Rinaldi L (2008). GIS modeling for canine dirofilariosis risk assessment in central Italy. Geospat Health.

[23] Simón L, Afonin A, López-Díez LI, González-Miguel J, Morchón R, Carretón E (2014). Geo-environmental model for the prediction of potential transmission risk of *Dirofilaria* in an area with dry climate and extensive irrigated crops. The case of Spain. Vet Parasitol.

[24] Lee AC, Atkins CE (2010). Understanding feline heartworm infection: disease, diagnosis, and treatment. Top Companion Anim Med.

[25] Berdoulay P, Levy JK, Snyder PS, Pegelow MJ, Hooks JL, Tavares LM (2004). Comparison of serological tests for the detection of natural heartworm infection in cats. J Am Anim Hosp Assoc.

[26] Venco L, Marchesotti F, Manzocchi S (2015). Feline heartworm disease: A ’Rubik’s-cube-like’ diagnostic and therapeutic challenge. J Vet Cardiol.

[27] Ryan GR, Newcomb KM. Prevalence of feline heartworm disease: a global review. In: Sol MD, Knight DH, editors. Proceedings of the heartworm symposium ‘95. Vatavia: American Heartworm Society; 1995. p. 79-86.

[28] Venco L, Genchi M, Genchi C, Gatti D, Kramer L (2011). Can heartworm prevalence in dogs be used as provisional data for assessing the prevalence of the infection in cats?. Vet Parasitol.

[29] Kramer L, Genchi C (2002). Feline heartworm infection: serological survey of asymptomatic cats living in northern Italy. Vet Parasitol.

